# Dielectrophoresis as a Tool to Reveal the Potential Role of Ion Channels and Early Electrophysiological Changes in Osteoarthritis

**DOI:** 10.3390/mi12080949

**Published:** 2021-08-11

**Authors:** Rula Abdallat, Emily Kruchek, Csaba Matta, Rebecca Lewis, Fatima H. Labeed

**Affiliations:** 1 Department of Biomedical Engineering, Faculty of Engineering, The Hashemite University, P.O. Box 330127, Zarqa 13133, Jordan; rulag@hu.edu.jo; 2 Centre for Biomedical Engineering, Department of Mechanical Engineering Sciences, University of Surrey, Guildford GU2 7XH, Surrey, UK; e.j.kruchek@surrey.ac.uk; 3 Department of Anatomy, Histology and Embryology, Faculty of Medicine, University of Debrecen, Nagyerdei krt 98, 4032 Debrecen, Hungary; matta.csaba@med.unideb.hu; 4 School of Veterinary Medicine, Faculty of Health and Medical Sciences, University of Surrey, Guildford GU2 7AL, Surrey, UK

**Keywords:** dielectrophoresis, DEP, ion channels, osteoarthritis, chondrocytes, detection

## Abstract

Diseases such as osteoarthritis (OA) are commonly characterized at the molecular scale by gene expression and subsequent protein production; likewise, the effects of pharmaceutical interventions are typically characterized by the effects of molecular interactions. However, these phenomena are usually preceded by numerous precursor steps, many of which involve significant ion influx or efflux. As a consequence, rapid assessment of cell electrophysiology could play a significant role in unravelling the mechanisms underlying drug interactions and progression of diseases, such as OA. In this study, we used dielectrophoresis (DEP), a technique that allows rapid, label-free determination of the dielectric parameters to assess the role of potassium ions on the dielectric characteristics of chondrocytes, and to investigate the electrophysiological differences between healthy chondrocytes and those from an in vitro arthritic disease model. Our results showed that DEP was able to detect a significant decrease in membrane conductance (6191 ± 738 vs. 8571 ± 1010 S/m^2^), membrane capacitance (10.3 ± 1.47 vs. 14.5 ± 0.01 mF/m^2^), and whole cell capacitance (5.4 ± 0.7 vs. 7.5 ± 0.3 pF) following inhibition of potassium channels using 10 mM tetraethyl ammonium, compared to untreated healthy chondrocytes. Moreover, cells from the OA model had a different response to DEP force in comparison to healthy cells; this was seen in terms of both a decreased membrane conductivity (782 S/m^2^ vs. 1139 S/m^2^) and a higher whole cell capacitance (9.58 ± 3.4 vs. 3.7 ± 1.3 pF). The results show that DEP offers a high throughput method, capable of detecting changes in membrane electrophysiological properties and differences between disease states.

## 1. Introduction

Articular cartilage is a tissue covering synovial joints and is responsible for load distribution across the joints [1]. The structure and function of the cartilage is controlled by chondrocytes, which are responsible for regulating and maintaining the cartilaginous extracellular matrix (ECM) [2,3]. Therefore, an imbalance in function or damage to these cells will cause changes to the ECM, leading to failure of cartilage function and the onset of degenerative diseases such as osteoarthritis (OA). OA is a common joint disorder that is characterized by pain and a restricted range of motion [4,5]. To date, no permanent cure for OA is available, and treatments given are limited to lifestyle changes and pain management [6]. This is due to the fact that OA is diagnosed by a history of pain and radiographic changes [6,7]. Currently, there is emerging interest to understand the role of chondrocytes and their mechanisms in cartilage repair and cartilage degeneration [2,8]. Progression of OA has been associated with changes in ECM composition and structure [9], resulting in an altered chondrocyte volume regulation which may be caused by changes in the membrane potential that is controlled by ion fluxes across the plasma membrane [10,11]. As a consequence, electrophysiological analysis may provide a new perspective to understanding OA progression.

While the most commonly used disease biomarkers are large molecules such as proteins, many diseases are associated with changes in cellular electrophysiology which can often act as a precursor to gene expression or protein production, allowing electrophysiological differences between healthy and pathological cell phenotypes to be used as a label-free biomarker [12,13]. Interest in ion channels as potential biomarkers of disease is increasing due to their involvement in major aspects of cell biology [14,15]. Ion channels are considered of vital importance in the cell membrane, as they control the membrane potential by coupling cell metabolism to electrical activity, and cytosolic ion concentration [16]. Chondrocytes contain a variety of ion channels, transporters, and pumps, collectively referred to as the channelome [17,18,19]. The synthesis and secretion of ECM proteoglycans and proteins, as well as mechanotransduction, apoptosis, cell volume regulation, and chondrogenesis are controlled by sequences of events activated by the precisely regulated dynamics of certain ions in the cytoplasm, in particular Ca^2+^ and K^+^ [7,20,21,22]. However, there is a particular scarcity of information on the activity and expression of ion channels mediating Ca^2+^ and K^+^ fluxes across the chondrocyte plasma membrane, and on their potential role in OA. Chondrocyte function is dependent on how ion channels operate related to the resting membrane potential and electrical properties of the membrane and cytoplasm [10,23,24]. Alterations in the membrane potential have been associated with ion channel activation, which may affect chondrocyte metabolic activity [11,25]. Therefore, measuring the electrophysiological changes after altering ionic homeostasis by inhibiting and/or blocking certain ion channels may significantly advance understanding of inflammatory disease progression, such as OA [26]. OA chondrocytes have a different response to osmotic changes compared to healthy chondrocytes and therefore can produce an altered pattern in ECM synthesis [27], increased expression levels of BK channels [15], and inhibited proliferation following blockage of voltage-gated K^+^ channels [28].

However, the ability to link between electrophysiology and pathophysiology is technically challenging due to the limitations of conventional methodology, such as difficulty in obtaining recordings from a large number of individual cells, and associated challenges with in vitro studies [29]. While traditional methods of cell electrophysiology, such as the gold standard patch clamp, can offer insights to detect changes in cellular properties [22,30,31,32], the method is highly technical and has a very low throughput [33,34]. Thus, the ability to detect such changes at high throughput is of great importance, particularly for drug discovery or large-scale population screening [35]. This has led to the recent development of several automated electrophysiology platforms such as lipid-soluble fluorescent probes [36], impedance spectroscopy [37], and dielectrophoresis (DEP) [38] to provide high-quality, high content, and high-throughput data. DEP was first described in the 1950s, and is defined as the motion of a polarizable particle (such as a cell) suspended in a medium and subjected to a non-uniform alternating electric field [39]. The DEP response is sensitive to both the electric field frequency and the electrical properties of particles [40], such that examining the response at multiple frequencies allows the determination of the resting whole cell electrical properties. DEP has been widely used for characterization of cellular electrophysiology [12,41], including apoptosis studies [42] and circadian rhythms [34]. Recent developments in 3D DEP technology have enabled near-real-time assessment of cell electrophysiology using the DEPtech 3DEP platform (Labtech, Heathfield, UK) [40,43,44], which enables the user to take ensemble measurements of the passive electrical properties of non-excitable cells at speed (typically 20,000 cells are analyzed simultaneously over a 10 s period).

In this paper, we used real-time DEP analysis to characterize and determine the contribution of K^+^ channels to the electrophysiological properties of articular chondrocytes. Tetraethyl ammonium chloride (TEA), a well-known K^+^ channel inhibitor, was used to block K^+^ efflux in chondrocytes to see whether DEP can detect an alteration in cell electrophysiology near-real-time. We also aimed to identify whether differences in electrophysiological parameters between healthy and arthritic-induced chondrocytes, as detected by DEP, have the potential to be a biomarker for disease.

## 2. Materials and Methods

### 2.1. Theory

The analysis of cell motion over a range of frequencies of an applied voltage during DEP experiments has been used for many years to determine the electrophysiological properties of cells, such as membrane capacitance, surface charge, and resistive properties of cell membrane and cytoplasm [38]. The DEP for a spherical particle (to which we approximate a cell) is given by Equation (1):

(1) $$F_{DEP}=2\pi \varepsilon_{m}r^{3}Re\left[K\left(\omega \right)\right]\nabla E^{2}$$

where $\nabla E^{2}$ is the gradient of the strength of the applied electric field squared, *ε_m_* is the permittivity of medium, ω is the angular frequency, and *Re[K(ω)]* is the real part of the Clausius–Mossotti factor (CM). CM factor is dependent on the dielectric properties of the particle and medium as well as the frequency of the applied electric field; it is the sign of the CM factor that will determine whether the particle undergoes positive or negative DEP. The CM factor is given by the following equation:

(2) $$K\left(\omega \right)=\frac{\varepsilon_{p}^{*}-\varepsilon_{m}^{*}}{\varepsilon_{p}^{*}+2\varepsilon_{m}^{*}}$$

where $\varepsilon_{p}^{*}$ and $\varepsilon_{m}^{*}$ are the complex permittivity of particle and medium, respectively. The complex permittivity is given by Equation (3):

(3) $$\varepsilon^{*}=\varepsilon -j\frac{\sigma }{\omega }$$

where ε refers to the real permittivity, and σ is the conductivity of the material.

It can be seen from Equations (2) and (3) that the CM factor is a function the dielectric properties (permittivity and conductivity) of the particle and the suspending medium, and the applied electric field frequency.

### 2.2. Chemicals

Unless stated otherwise, all chemicals used in this study were purchased from Sigma-Aldrich (Poole, UK).

### 2.3. Cell Culture

Primary articular chondrocytes were sourced from the metacarpophalangeal or stifle joints of equines or canines, respectively. Chondrocytes were isolated as described previously [11]. Cells were cultured in Dulbecco’s Modified Eagle’s Medium (DMEM) with glutamine, supplemented with 10% fetal bovine serum, 100 units/mL penicillin, and 100 μg/mL streptomycin. Cells were cultured using standard conditions, a humidified incubator at 37 °C and 5% CO_2_/95% air. At 60–70% confluence, cells were detached using 0.25% trypsin–EDTA, then sub-cultured.

### 2.4. OA-Induced Cell Model

Once chondrocyte cultures were established, cells were split into two groups: (i) untreated control which was maintained as described above, and (ii) stimulated cells which were cultured in DMEM as above plus TNF-α and IL-1β (both at 10 ng/mL). Chondrocytes were cytokine-stimulated for up to one week. Cells were used in experiments up to the 2nd passage.

### 2.5. Pharmacological Treatment

TEA (10 mM), a potassium channel blocker, was added to (i) chondrocytes in supplement free culture media and incubated for 5 min before washing and resuspending cells in DEP media (referred to as chronic treatment), or (ii) directly to chondrocytes suspended in DEP media just prior to performing characterization experiments (referred to as acute treatment).

### 2.6. Sample Preparation

DEP iso-osmotic experimental media consisting of deionized water mixed with 8.5% sucrose (*w*/*v*), 0.3% dextrose (*w*/*v*), 100 μM CaCl_2_, and 250 μM MgCl_2_ was freshly prepared [40]. The medium conductivity was adjusted to 150 mS/m using phosphate-buffered saline (PBS), with [K^+^] to be ~5 mM, which would not affect the intracellular [K^+^] ≈ 150 mM. The conductivity was verified using a Jenway conductivity meter (VWR Jencons, Leicestershire, UK). Cells were prepared by washing twice at 200× *g* for 5 min then resuspended in DEP medium. Final cell concentration used was 1 × 10^6^ cells/mL (±10%).

Cell radii were measured for each sample by capturing images of cells on a haemocytometer using an AVT Dolphin F145B digital camera (Allied Vision Technologies, Stadtroda, Germany) connected to a Nikon Eclipse microscope and a PC. Images were analysed using ImageJ (National Institute of Mental Health, Bethesda, MD, USA) image analysis software.

### 2.7. Dielectrophoretic Experiments and Analysis

After suspending cells in DEP media, cell suspensions were pipetted into 3DEP chips (DEP tech international, Heathfield, UK). The chips were then inserted into the 3DEP reader (DEP tech international, Heathfield, UK). Pin connections in the reader energized 20 wells in the chip with 20 different frequencies at a voltage of 10Vp-p for 30 s. The process was performed for equine cells (Control, Chronic, and Acute), as well as canine cells (cytokine treated and control cells). For each condition, four biological repeats were performed, and for each biological repeat, at least 3 technical repeats were performed. Raw data were then fitted into a mathematical model (single shell model) of the CM factor to extract the electrophysiological properties. Models producing an R value (Pearson correlation coefficient) of 0.9 or less were excluded [34,40]. All data are presented as the mean ± SD. Statistical analysis of the results was conducted using unpaired two-tailed *T*-test in Graphpad Prism version 9.0.0 for Windows (Graphpad Software, San Diego, CA, USA).

## 3. Results and Discussion

### 3.1. Role of Potassium Ions in Chondrocyte Electrophysiology

Given the central role of K^+^ homeostasis, and the wide variety of potassium channels in chondrocytes with a direct relevance to the OA phenotype [19,45], we focused on measuring the effect of K^+^ channel modulation. To this end, cells were treated with 10 mM of TEA (an inhibitor of K^+^ channels) to observe real-time changes in cell properties. TEA was added directly to chondrocytes suspended in DEP media (Acute); the extracted electrophysiological parameters were compared to untreated cells (Control) and cells incubated in culture medium containing the drug prior to resuspension in DEP media for experimentation (Chronic). The radii of treated and untreated cells were measured prior to performing DEP to identify any changes between the groups; cell radius measurements were reproducible for all experiments (*n* = 120 for each experiment). No significant difference was observed in the radii measurements between treated and untreated cells. The mean diameters for each group are listed in Table 1 and were used to model the dielectric behavior.

**Figure 1:**
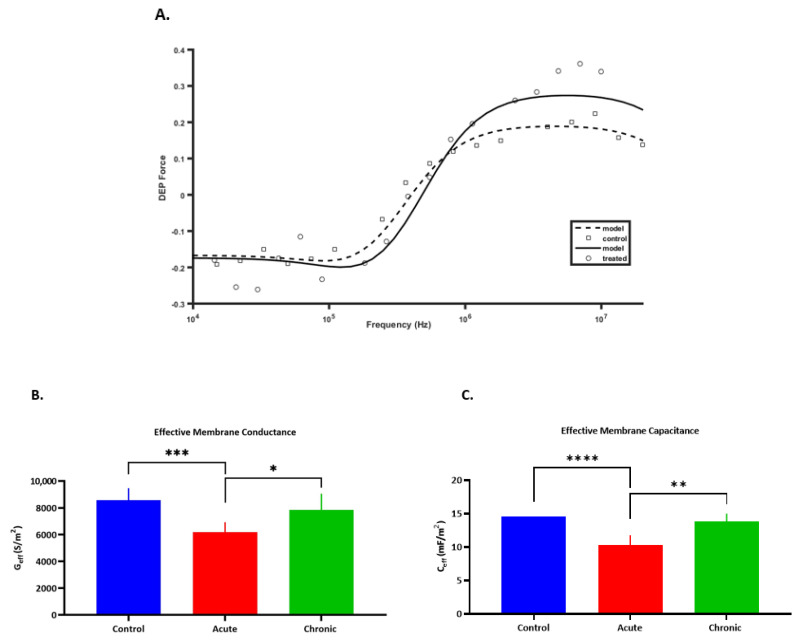
(**A**) An example of an electrophysiological DEP spectrum, derived from untreated (control) and TEA-treated chondrocytes. (**B**) Effective membrane conductance (G_eff_). (**C**) Effective membrane capacitance (C_eff_) for untreated (Control), 10 mM TEA treated in DEP (Acute), and treated in culture media (Chronic) chondrocytes. Each experiment had 4 biological repeats, and for each biological repeat at least three technical repeats were performed. Both values were derived by best fit line to dielectric spectra using the single shell model. Error bars represent standard deviation (SD). Asterisks denote significant differences (**** *p* ˂ 0.0001, *** *p* ˂ 0.001, ** *p* ˂ 0.01, * *p* ˂ 0.05).

DEP spectra (Figure 1A) were produced and modeled using the single shell model to determine electrophysiological properties based on changes in the light intensity [43]. Effective membrane conductance (G_eff_), effective membrane capacitance (C_eff_), and cytoplasmic conductivity (σ_cyt_) of each group were extracted and are summarized in Table 1.

Inhibiting K^+^ efflux caused a significant drop in membrane conductance values (G_eff_) for the Acute group (6191 S/m^2^ (*p <* 0.001)), in comparison to Control and Chronic samples that showed mean values of 8570 S/m^2^ and 7857 S/m^2^, respectively (Figure 1B). This decrease in G_eff_ may indicate attenuated K^+^ transport across the membrane [46], as membrane conductance reflects the net transport of ions across the cell membrane. Our measured values of membrane conductance for control cells (8570 S/m^2^) are in line with reported G_eff_ values for human chondrocytes which range between 7500 and 12,000 S/m^2^ [24,47].

A significant decrease was also observed in C_eff_ (Figure 1C) for the Acute group (10.3 ± 1.47 mF/m^2^; (*p <* 0.0001)), compared to Control (14.5 ± 0.01 mF/m^2^) and Chronic (13.9 ± 1.22 mF/m^2^). This decrease in effective membrane capacitance is related to the change in cell morphology and size [13]. Although there was no visible change in the cell radius for treated cells, a decrease in C_eff_ following treatment indicates membrane stretching (i.e., less folding of the cell membrane). Membrane capacitance values are in line with reported values for different cell types such as chondrocytes and lymphocytes, that are usually larger than 10 mF/m^2^ [24,48]. The membrane capacitance of human costal chondrocytes was reported to be around 9.4 mF/m^2^ using dielectric spectroscopy [47]. Furthermore, our data concur with the minimum value of lipid bilayer capacitance of ~8 mF/m^2^, with elevated values beyond this suggesting the presence of micro- and nanostructures on cell membrane, which affect the total cell membrane capacitance by increasing the surface area [49].

Moreover, the significant difference (*p* ˂ 0.05) between the Chronic and Acute groups for both G_eff_ and C_eff_ must be noted as it indicates the ability of DEP to detect electrophysiological changes directly once TEA was added to cells in DEP media in comparison to culturing cells in serum free culture media supplemented with TEA. Despite previous literature suggesting concentrations as low as 0.66 mM to be efficient on chondrocytes [50,51]. Data from the Chronic model suggest that longer incubation time in culture media or higher TEA concentration is required to show the TEA effects.

No statistical change was observed in σ_cyt_ following treatment, although a non-significant rise of approximately 10% in σ_cyt_ was detected in Chronic equine cells when compared to Control and Acute treatments. This small but non-significant increase is related to the increase in ion concentration as K^+^ channels are blocked by TEA, as K^+^ can no longer flow out of the cell [43].

In order to compare the values of membrane properties obtained by DEP with those measured by other techniques, whole cell capacitance was calculated [40] using values of C_eff_ described above, together with cell surface area obtained from measured cell radii. A significant drop (*p =* 0.0001) was observed in whole cell capacitance following acute TEA treatment, with treated cells exhibiting whole cell capacitance of 5.4 ± 0.7 pF in comparison to untreated cells of 7.5 ± 0.3 pF. The decrease in cell capacitance values following inhibiting K^+^ outward flux suggests a change in cell morphology. Reported values of cell capacitance of healthy human articular chondrocytes using patch clamping were found to be between 7.1 and 8.1 pF [1], which are in line with our results for healthy untreated cells (7.5 ± 0.3 pF).

Cell capacitance that results from the plasma membrane acting as a capacitor contributes in determining the time constant that controls how fast the membrane potential responds to changes in current caused by ionic flux [52]; our data showed that there was a reduction in chondrocyte response due to blockade of K^+^ efflux, which may lead to an altered membrane potential. Several studies [1,17,51,53] have used TEA to block outward currents in chondrocytes and have reported a demolished current after treatment. Patch clamp data showed that TEA treatment affects outward currents in primary murine chondrocytes causing membrane potential to depolarize to about −42 mV in comparison to the RMP value of −46.7 mV [54]. Another study [55] found that rat articular chondrocytes became hyperpolarized when placed in a K^+^ hyperosmotic medium; on the contrary, the same cell type experienced depolarization (−26 ± 4.0 mV) when treated with 10 mM TEA as compared to the RMP (−42.7 ± 2.0 mV). Maintaining cell volume is crucial for cell survival and function, and it is thought that the membrane potential is likely to be a key player in volume maintenance in many cells [56]. Therefore, changes in K^+^ efflux across the plasma membrane would also affect the membrane potential, which depends on the efficiency of ion channels. Note that our DEP values for whole cell capacitance of equine chondrocytes were smaller than reported values using patch clamp techniques (23.8 ± 2.8 pF) [51]; this can potentially be explained by the small numbers of cells used in patch clamping in comparison to the cell numbers used in DEP technique; moreover, there is a tendency in patch clamping to pick the largest cells that are easier to analyze and therefore the mean capacitance values can vary significantly [40].

Ion channels play an essential role in the activity of many intracellular processes such as mechanotransduction and biosynthesis in cells [8,23,57]. By regulating ionic flux, which is responsible for the magnitude and direction of cellular membrane potential [1,16], changes in ion concentration homeostasis can lead to alterations in resting membrane potential affecting cell functions such as cellular metabolism, phenotype, and volume regulation [23,56,58]. One of the benefits of electrophysiological measurements is the ability to study the effect of drugs such as channel blockers, which disrupt bioprocesses such as mechanotransduction and biosynthesis [23,57]. Based on our results, we found that DEP—a rapid and label-free technique—was able to detect subtle changes in electrophysiological properties in chondrocytes following K^+^ efflux inhibition. Our measured values of membrane conductance and cell capacitance are in line with previous studies performed using the conventional patch clamping techniques.

### 3.2. Electrophysiological Differences between Healthy and Arthritic Chondrocytes

Biomarkers can be of great importance in identifying changes in characteristics, origin, and cell fate, and in detecting electrophysiological differences between healthy and OA chondrocytes [59,60]. In order to examine electrophysiological differences between healthy and OA chondrocytes, cells from our in vitro arthritis model were characterized using DEP. As previously described, the radii of control and OA-induced cells were measured prior to performing DEP to determine any size difference between the two groups. Cell radius measurements were reproducible for all experiments (*n* = 120 for each experiment). A significant, 20% increase in cell radius was noted in arthritic chondrocytes (*p* < 0.0001), indicating a change in volume and cell surface area between induced OA and control cells. It has been suggested that the volume and morphology of chondrocytes have a role in controlling cell phenotype; therefore, any change in the above might cause the production of very different extracellular proteins leading to a weak and mechanically inadequate ECM [8]. The mean diameters for each group (Table 2) were used to model the dielectric behavior.

**Figure 2:**
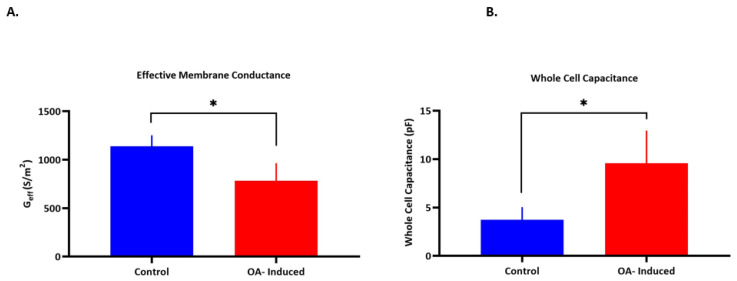
(**A**) Effective membrane conductance (G_eff_). (**B**) Whole cell capacitance for control and OA-induced canine chondrocytes. Error bars represent SD. Asterisks denotes significant differences (* *p* ˂ 0.05).

Additionally, DEP spectra were collected and analyzed to extract the electrophysiological properties (Table 2). A significant decrease (*p =* 0.0159) was observed in G_eff_ between OA induced and healthy chondrocytes, with values of 782 S/m^2^ compared to 1139 S/m^2^, respectively (Figure 2A). Membrane conductance extracted by DEP represents the electric conduction both through and around the cell membrane, and reflects ion channel activity on the cell membrane [46,61]. Therefore, the decrease in membrane conductance may be explained by the decrease in ion transport across the membrane. Moreover, whole cell capacitance (Figure 2B) increased significantly (*p =* 0.0175) in the arthritic cells (9.58 ± 3.4 pF) in comparison to control cells (3.7 ± 1.3 pF). This increase in capacitance can be related to the changes and differences in membrane composition and folding [62]. On the other hand, there was no significant change in C_eff_ between arthritic cells (6.92 ± 3.5 mF/m^2^) and the control (7.51 ± 2.6 mF/m^2^); this is interesting given the different radii of the cells and suggests no change in the membrane morphology accompanying the change in cell size—for example, cell membrane smoothing as they swell. Furthermore, no statistical difference was observed in cytoplasmic conductivity (σ_cyt_). Bertram et al. [7] reported electrophysiological differences between healthy and osteoarthritic synovial fluid cells using patch clamping technique; OA cells had higher capacitance than their healthy counterparts. The cell membrane potential, which results from the balance of ion changes through ion channels on the plasma membrane, has a significant impact on the ionic fluxes and thus may influence chondrocyte metabolism. OA human chondrocytes showed an upregulated gene expression profile of K^+^ channels, and a significantly higher cell capacitance (≈37.93 pF) in comparison to their healthy counterparts (≈21.56 pF) [63]. Moreover, OA chondrocytes were documented to have a hyperpolarized membrane potential (−26 ± 4 mV vs. −23 ± 2.9 mV) in comparison to healthy cells [27,64]. This change in membrane potential was also reported by inhibiting K^+^ channels due to the altered decrease in ion flux [55]. K^+^ channels in particular play a major role in generating the membrane potential of most animal cells [1]. Previous studies showed that TEA decreased chondrocyte proliferation [28], increased the RMP [65], and reduced the secretion of ECM components [28]. Moreover, the expression levels of these channels was shown to be altered in OA, which suggest their involvement in disease progression, as changes in ion flux would cause an abnormal membrane potential [23,45].

## 4. Conclusions

There is a need for fast, accurate, reliable, and label-free techniques to assess changes in cell electrophysiology; this would enable a greater understanding of drug interaction mechanisms and disease progression. In this study, the DEP responses of chondrocytes were investigated in order to study differences in dielectric properties between healthy and arthritic models, as well as the ability of DEP to detect near-real-time alterations in K^+^ efflux. Our data indicate that DEP was able to detect rapid changes in K^+^ efflux following ion channel blocking using TEA. Moreover, we demonstrated that DEP response was different between healthy and arthritic chondrocytes. The arthritic cell model showed a decrease in membrane conductance, which is related to a decrease in ionic conduction. Conventional cell assays suffer from low throughput, high cost, and are labor intensive. DEP serves as a high-throughput and label-free technique that does not require complicated skills to perform electrophysiological measurements. The ability to easily determine the electrophysiological properties using DEP may be used in characterizing and identifying ion channel contribution in chondrocytes, as well as its potential to reveal early changes in cellular properties before symptomatic OA presents, which could help us develop better/more targeted pharmaceuticals.

## Figures and Tables

**Table 1:** Electrophysiological parameters for chondrocytes derived from DEP data: (a) healthy untreated cells (Control); (b) chondrocytes incubated for 5 min. in supplement free culture media containing 10 mM TEA before washing and resuspending in DEP media (Chronic); (c) chondrocytes suspended directly in DEP media containing 10 mM TEA (Acute). Data presented are mean ± SD. Asterisks denote significant differences between treated and untreated cells (* *p* ˂ 0.05).

	r (μm)	Membrane Conductance	Membrane Capacitance	Cytoplasm Conductivity
		(G_eff_) (S/m^2^)	(C_eff_) (mF/m^2^)	(σcyt) (S/m)
a-Control	6.40 (±0.12)	8571 (±1010)	14.5 (±0.01)	0.26 (±0.01)
b-Chronic	6.58 (±0.18)	7857 (±1190)	13.9 (±1.22)	0.29 (±0.03)
c-Acute	6.47 (±0.07)	6191 (±738) *	10.3 (±1.47) *	0.27 (±0.05)

**Table 2:** Electrophysiological parameters for OA induced chondrocytes derived from DEP data. Data presented are mean ± SD. Asterisks denote significant differences between treated and untreated cells (* *p* ˂ 0.05).

	r (μm)	Membrane Conductance	Membrane Capacitance	Cytoplasm Conductivity
		(G_eff_) (S/m^2^)	(C_eff_) (mF/m^2^)	(σ_cyt_) (S/m)
a-Control	6.3 (±0.82)	1139 (±112)	7.51 (±2.6)	0.22 (±0.06)
b-OA-induced	8.5 (±0.97) *	782 (±183) *	6.92 (±3.5)	0.24 (±0.06)

## Data Availability

Data can be provided upon request.
